# Supplementary material for: Application of machine learning algorithms to identify serological predictors of COVID-19 severity and outcomes
Source: Commun Med (Lond). 2024 Nov 26;4:249. doi: 10.1038/s43856-024-00658-w (PMC11599591; doi:10.1038/s43856-024-00658-w)

Supplementary Table 1. Categorization of COVID-19 severity based on the WHO ordinal scale.

WHO Severity Score		
**Patient State**	**Description**	**Max Score**
Non-hospitalized	No limitation of activities	1
	Limitation of activities	2
Hospitalized-Moderate	Hospitalized, no oxygen required	3
	Oxygen by mask or nasal prongs	4
Hospitalized-Severe	Non-invasive ventilation or high-flow oxygen	5
	Intubation and mechanical ventilation	6
	Ventilation + organ support (e.g., RRT, pressors, ECMO)	7
Hospitalized-Deceased	Death	8

Supplementary Table 2. Comparison of cytokines and chemokines among hospitalized COVID-19 patients at enrollment (n=15 moderate, 20 severe, and 11 deceased) by Welch's ANOVA.

Cytokine/Chemokine	Moderate vs. Severe	Moderate vs. Deceased	Severe vs. Deceased
**Eotaxin**	0.55	0.61	0.31
**Eotaxin-3**	0.30	0.41	0.94
**GM-CSF**	0.42	0.38	0.95
**IFN-α2a**	0.11	0.68	0.07
**IFN-β**	0.54	0.20	0.49
**IFN-γ**	0.80	0.73	0.93
**IL-10**	0.75	0.58	0.30
**IL-12p70**	0.03	0.36	0.32
**IL-13**	0.85	0.83	0.94
**IL-15**	0.08	0.005	0.32
**IL-16**	<0.001	0.001	0.90
**IL-17A**	0.17	0.07	0.46
**IL-18**	0.16	0.11	0.81
**IL-1RA**	0.17	0.07	0.76
**IL-1α**	0.20	0.44	0.48
**IL-1β**	0.20	0.88	0.21
**IL-2**	0.16	0.43	0.57
**IL-23**	0.97	0.66	0.64
**IL-2Ra**	0.19	0.10	0.66
**IL-4**	0.07	0.19	0.48
**IL-5**	0.06	0.29	0.47
**IL-6**	0.02	0.009	0.48
**IL-7**	0.007	0.10	0.20
**IL-8**	0.02	<0.001	0.12
**IP-10**	0.57	0.16	0.31
**MCP-1**	0.03	<0.003	0.17
**MCP-2**	0.84	0.15	0.15
**MCP-4**	0.58	0.84	0.77
**MDC**	0.35	0.31	0.81
**MIP-1α**	0.37	0.31	0.87
**MIP-1β**	0.03	0.30	0.20
**TARC**	0.90	0.11	0.28
**TNF-α**	0.12	0.03	0.53
**TNF-β**	0.83	0.47	0.58
**VEGF**	0.02	0.29	0.50

Supplementary Figure 1. Distribution of samples collected by days post-enrollment among non-hospitalized and hospitalized COVID-19 patients. Dashed lines represent the mean ± SD days post-enrollment of non-hospitalized patients. Box plot of the days post-enrollment to death among subsequently deceased hospitalized patients.

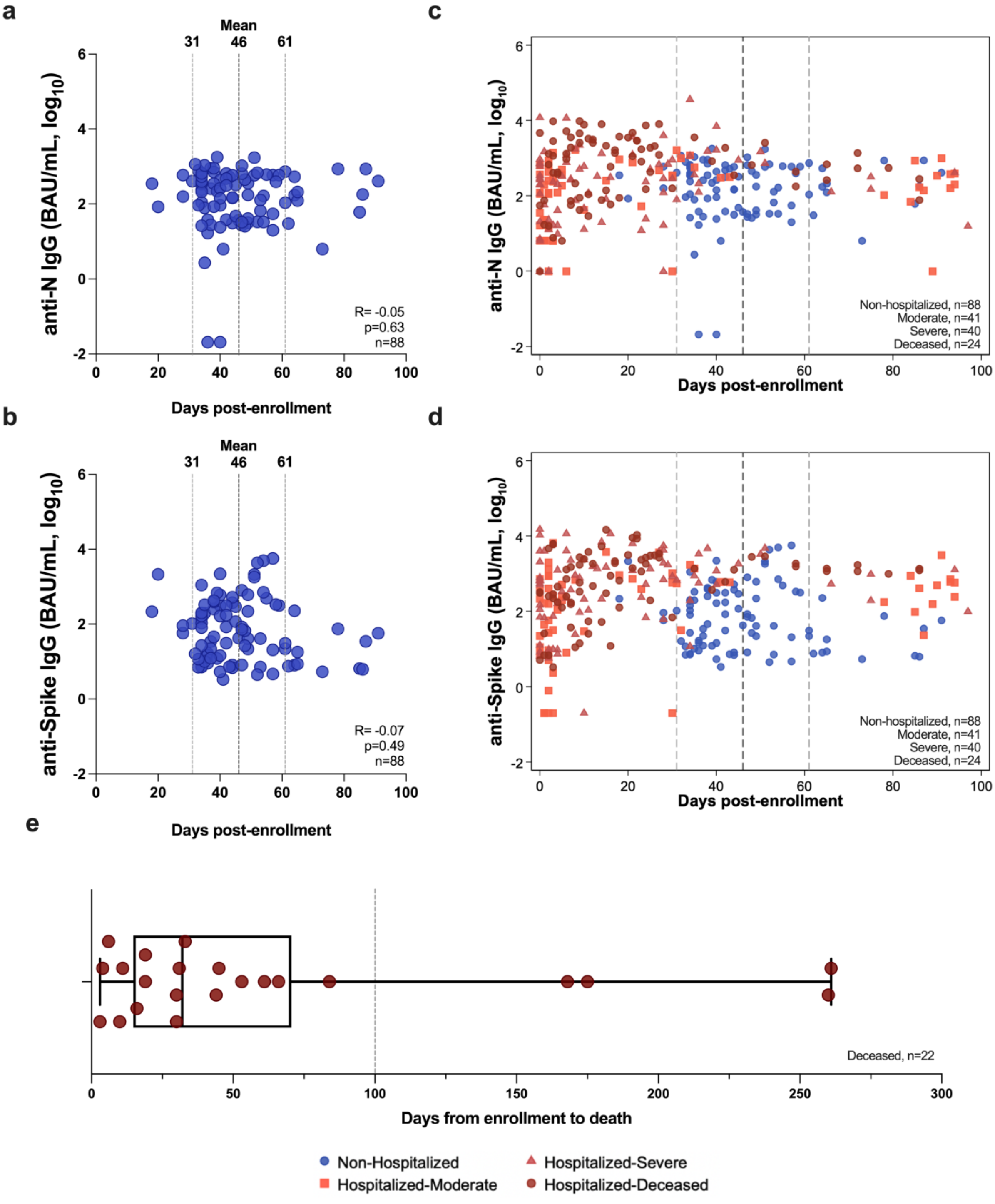

Supplementary Figure 2. HTetZ/SW1 HEK-293 cells express Spike protein in response to doxycycline (DCC). The expression of Spike protein on HTetZ/SW1 HEK-293 cells after overnight treatment with DCC was confirmed by flow cytometry using (a) a commercial antibody (anti-SARS-CoV-2 2019-nCoV spike S2 antibody, Sino Biological 40590-D001) and (b) purified IgG from anti-Spike positive patient plasma (aSPPP; n=3). Anti-mouse BV421 and anti-human Alexa Fluor 488 IgG were used as secondary antibodies, respectively. HTetZ HEK-293 cells, which do not express Spike after DCC treatment, as well as cells treated with secondary antibodies alone (Sec. Ab Alone), were used as negative controls. (c) The gating strategy used for flow cytometry analysis is shown, with the geometric mean (GM) representing the Mean Fluorescence Intensity (MFI). GM and cell percentages (%) for commercial antibody and aSPPP IgG were calculated based on the Scatter gate. Spike surface expression was detected in 94.8% of DCC-treated HtetZ/SW1 HEK-293 cells using 2 µg of purified IgG from aSPPP, with an AF488 MFI of 193312, as shown in (c). Refer to Supplementary Table 3 for the MFI and cell percentage values across all conditions.

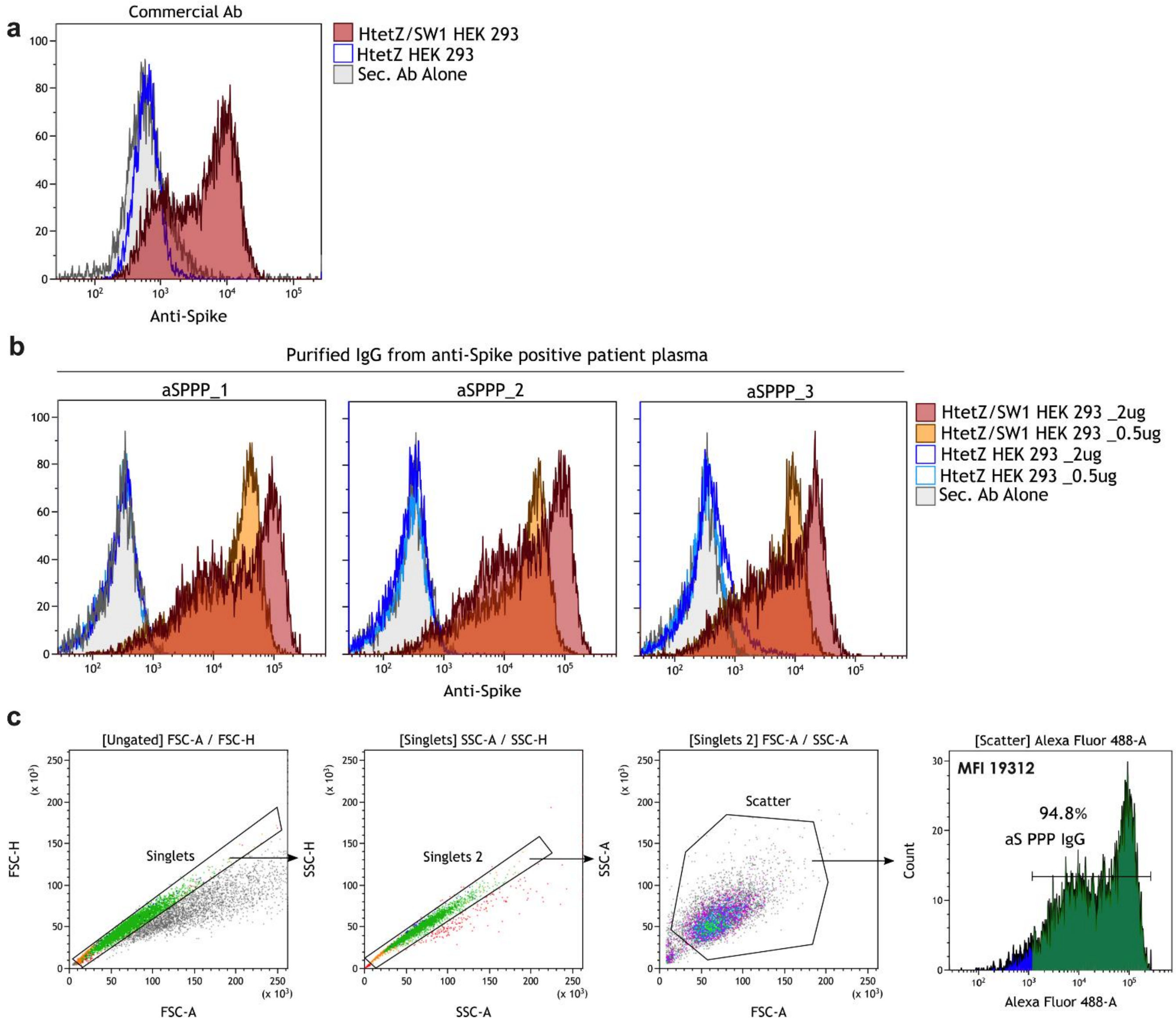

Supplemental Table 3. Mean Fluorescence Intensity (MFI) and cell percentage values. Refer to the gating strategy depicted in Supplemental Figure 2C for further details.

				Anti-Spike			
				aS PPP IgG		SN 40590	
Cell Target	Antibody	Group	ug Ab/well	AF488 MFI	%	BV421 MFI	%
HtetZ HEK293	Sino Biological 40590-D001	Commercial Ab	0.5	---	---	464	2.1
Htetz/CG145 (SpikeW1) HEK293	Sino Biological 40590-D001	Commercial Ab	0.5	---	---	3484	73.3
Htetz/CG145 (SpikeW1) HEK293	BV421 Sec antibody_ Biolegend 405317	Commercial Ab-Sec.Ab	0.5	---	---	350	1.6
HtetZ HEK293	anti-Spike PPP purified IgG	High_1	0.5	98	0.1	---	---
HtetZ HEK293	anti-Spike PPP purified IgG	High_1	2	99	0.3	---	---
Htetz/CG145 (SpikeW1) HEK293	anti-Spike PPP purified IgG	High_1	0.5	12367	92.8	---	---
Htetz/CG145 (SpikeW1) HEK293	anti-Spike PPP purified IgG	High_1	2	18286	94.2	---	---
HtetZ HEK293	anti-Spike PPP purified IgG	High_2	0.5	109	0.2	---	---
HtetZ HEK293	anti-Spike PPP purified IgG	High_2	2	113	0.2	---	---
Htetz/CG145 (SpikeW1) HEK293	anti-Spike PPP purified IgG	High_2	0.5	13442	93.2	---	---
Htetz/CG145 (SpikeW1) HEK293	anti-Spike PPP purified IgG	High_2	2	19312	94.8	---	---
HtetZ HEK293	anti-Spike PPP purified IgG	Medium	0.5	121	0.3	---	---
HtetZ HEK293	anti-Spike PPP purified IgG	Medium	2	120	0.4	---	---
Htetz/CG145 (SpikeW1) HEK293	anti-Spike PPP purified IgG	Medium	0.5	3333	80.7	---	---
Htetz/CG145 (SpikeW1) HEK293	anti-Spike PPP purified IgG	Medium	2	5524	85.2	---	---
Htetz/CG145 (SpikeW1) HEK293	AF488 Sec Antibody_Thermo Fisher A11013	High_1/_2/Medium- Sec.Ab	0.5	109	0.4	---	---
Htetz/CG145 (SpikeW1) HEK293	---	Unstained	---	63	0.2	321	1.4

Supplementary Figure 3. Mucosal secretory IgA (sIgA) responses against beta coronaviruses in nasopharyngeal and oropharyngeal samples among hospitalized COVID-19 patients at enrollment.

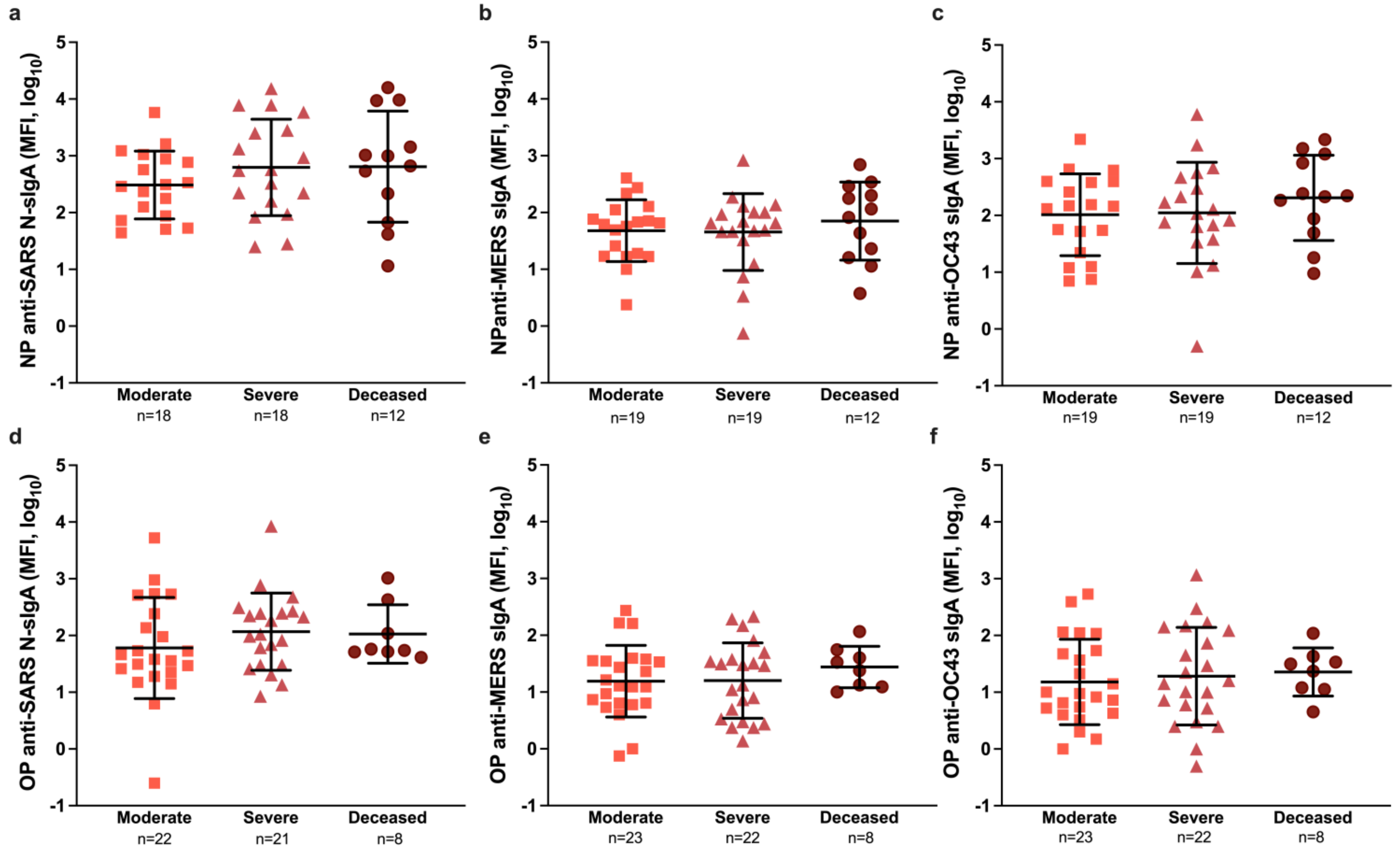

Supplementary Figure 4. Mucosal IgG responses against beta coronaviruses nasopharyngeal and oropharyngeal samples among hospitalized COVID-19 patients at enrollment.

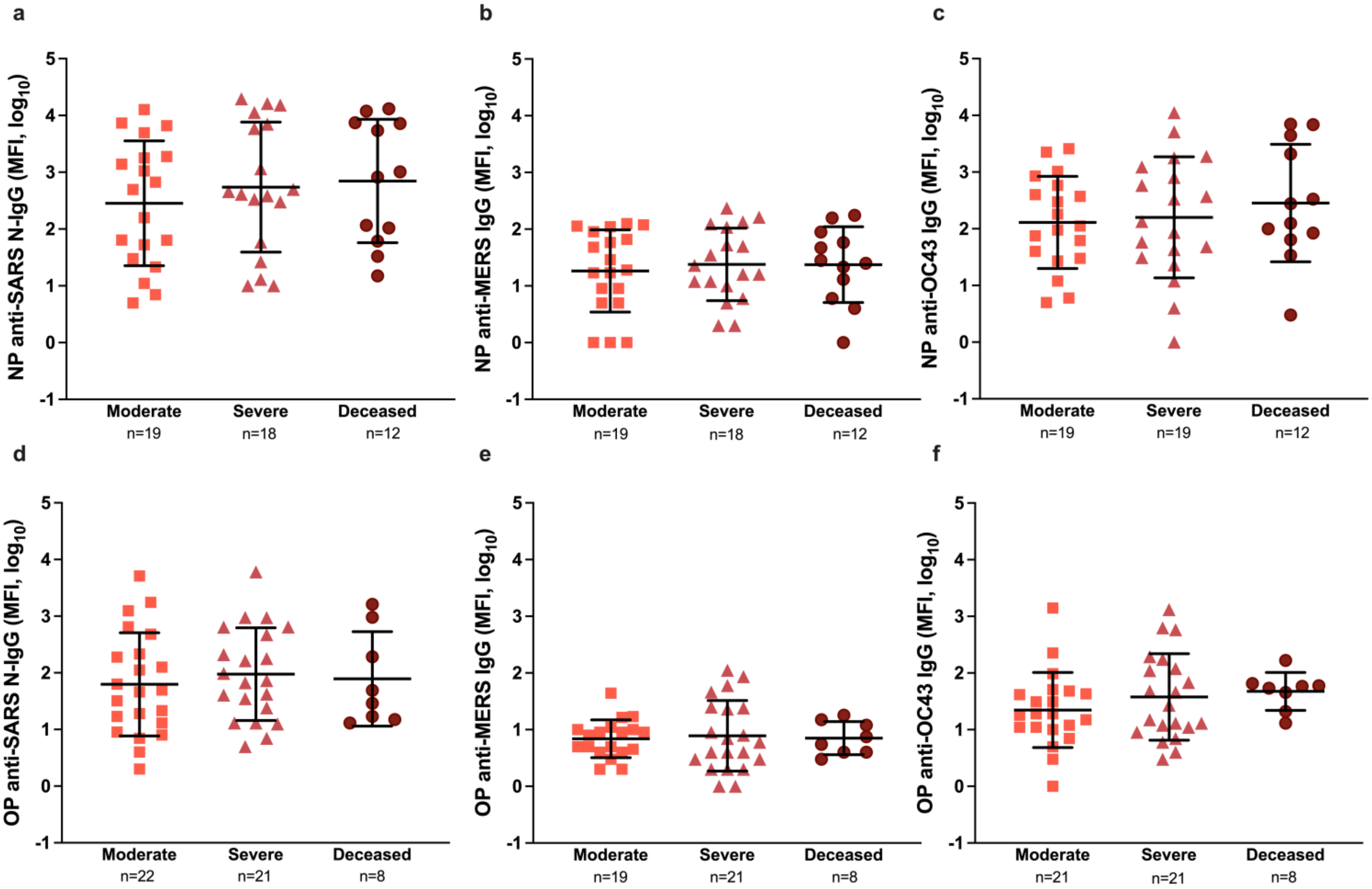

Supplementary Figure 5. Sex differences in antibody responses among non-hospitalized and hospitalized COVID-19 patients at 1-month post-enrollment (1 MPE).

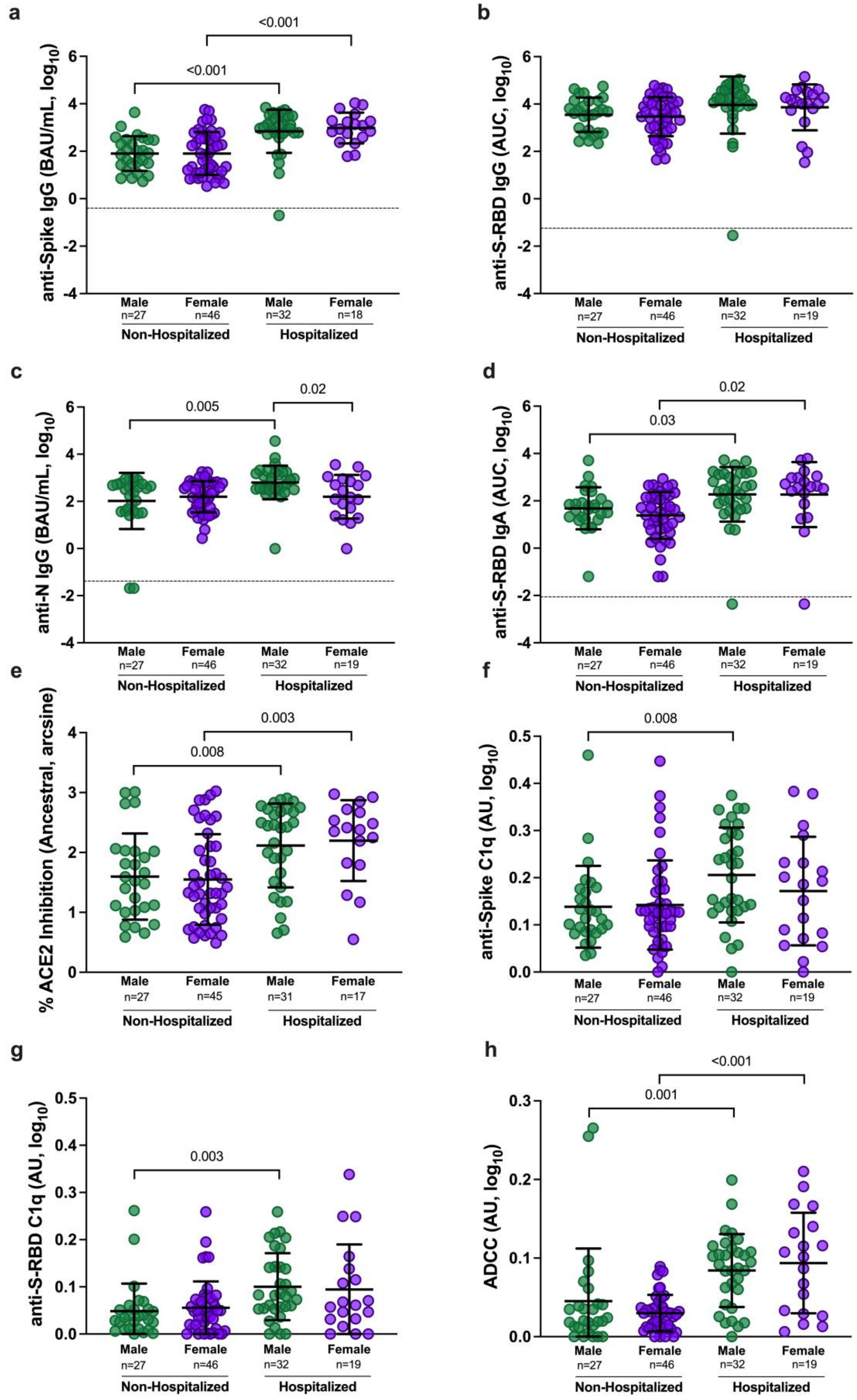

Supplementary Figure 6. Age-associated differences in antibody responses against SARS-CoV-2 between non-hospitalized and hospitalized COVID-19 patients at 1-month post-enrollment (MPE).

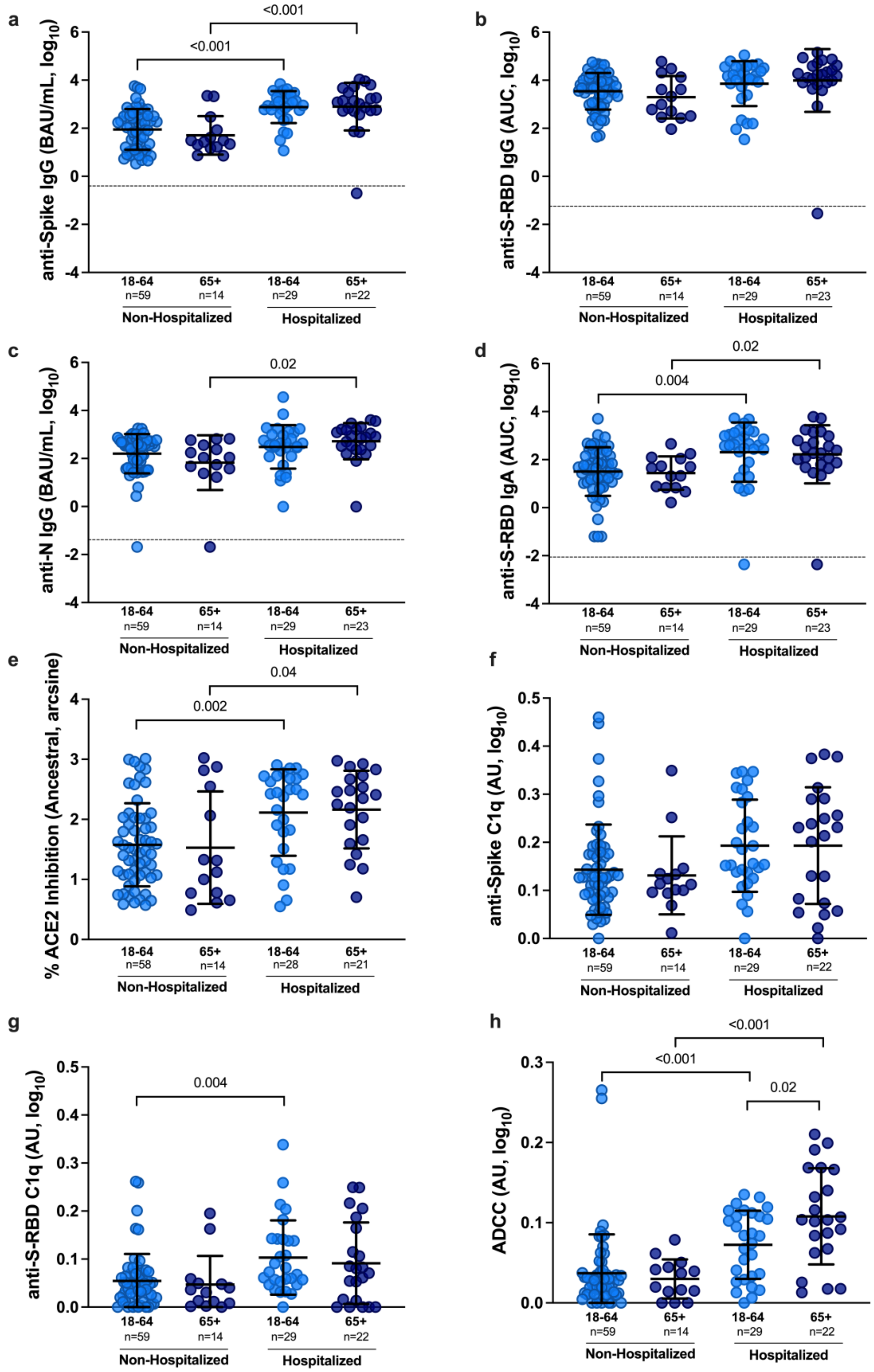

Supplementary Figure 7. ACE2-inhibition antibody responses against SARS-CoV-2 variants among hospitalized COVID-19 patients at enrollment.

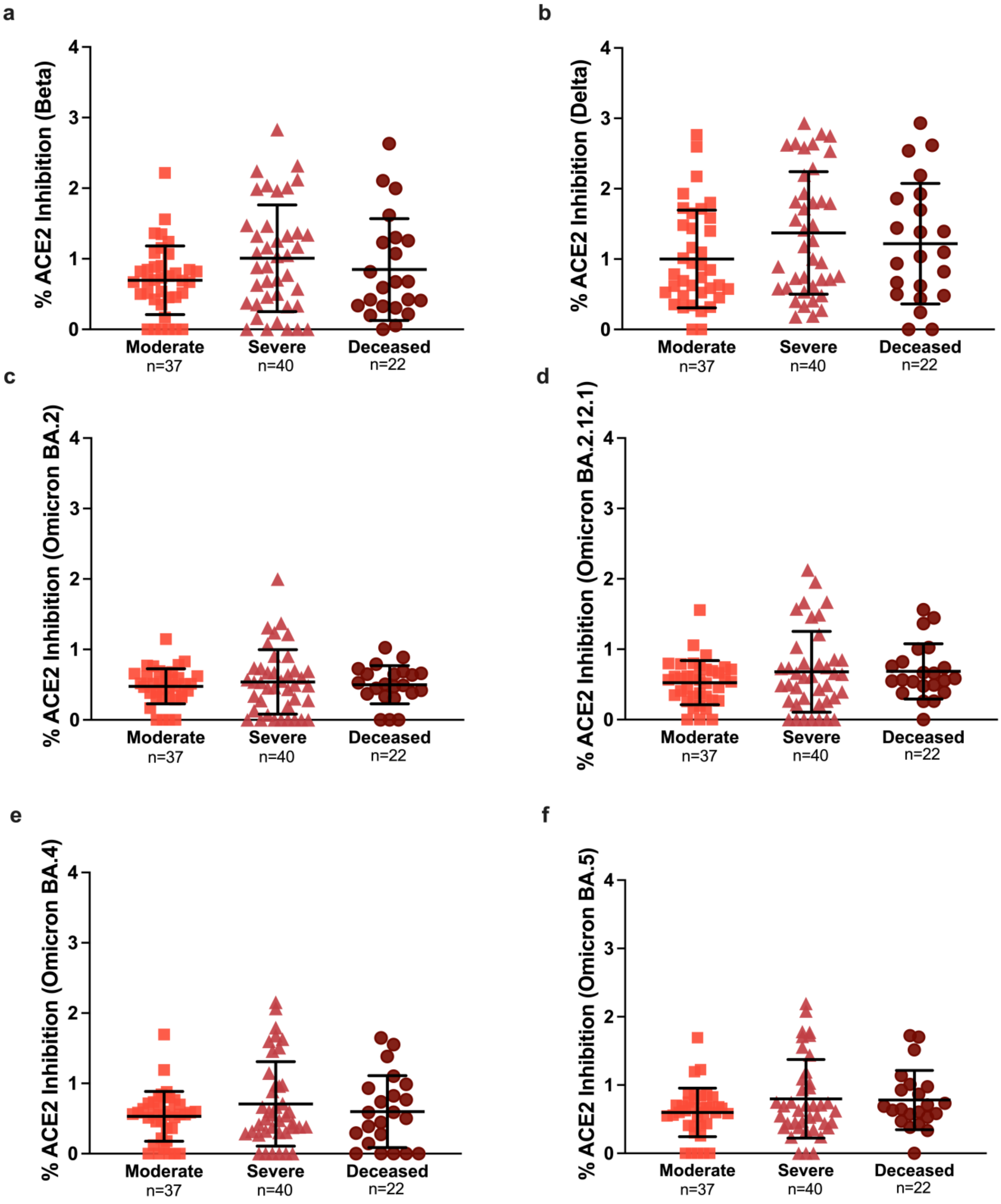

Supplementary Figure 8. Spearman correlation of plasma complement anti-spike and spike receptor binding domain (S-RBD) C1q levels with binding antibodies and subclasses of spike antibodies.

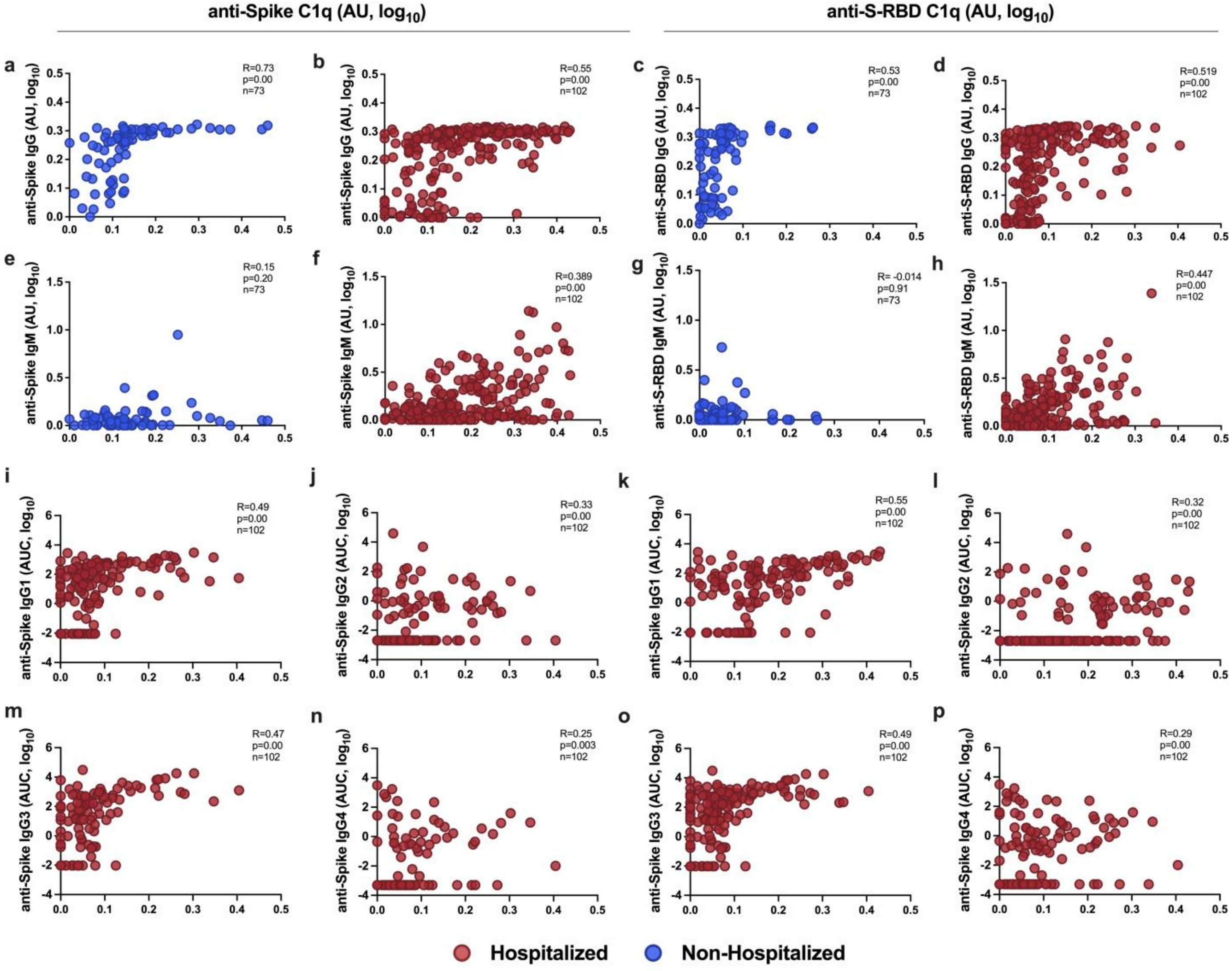

Supplementary Figure 9. Model performance metrics at a cutoff of 0.46 for the random forest intubation models at enrollment (n=45 intubated of 98 hospitalized with complete data). Receiver operating characteristic (ROC) curve for the model with sociodemographic and serological measures with an area under the curve (AUC) value of 0.74. For the random forest model with sociodemographic and serological measures, partial dependence plots and bivariate dependence plots were used to assess the associations of the top four variables with the predicted probability of intubation among hospitalized COVID-19 patients, while controlling for all other variables.

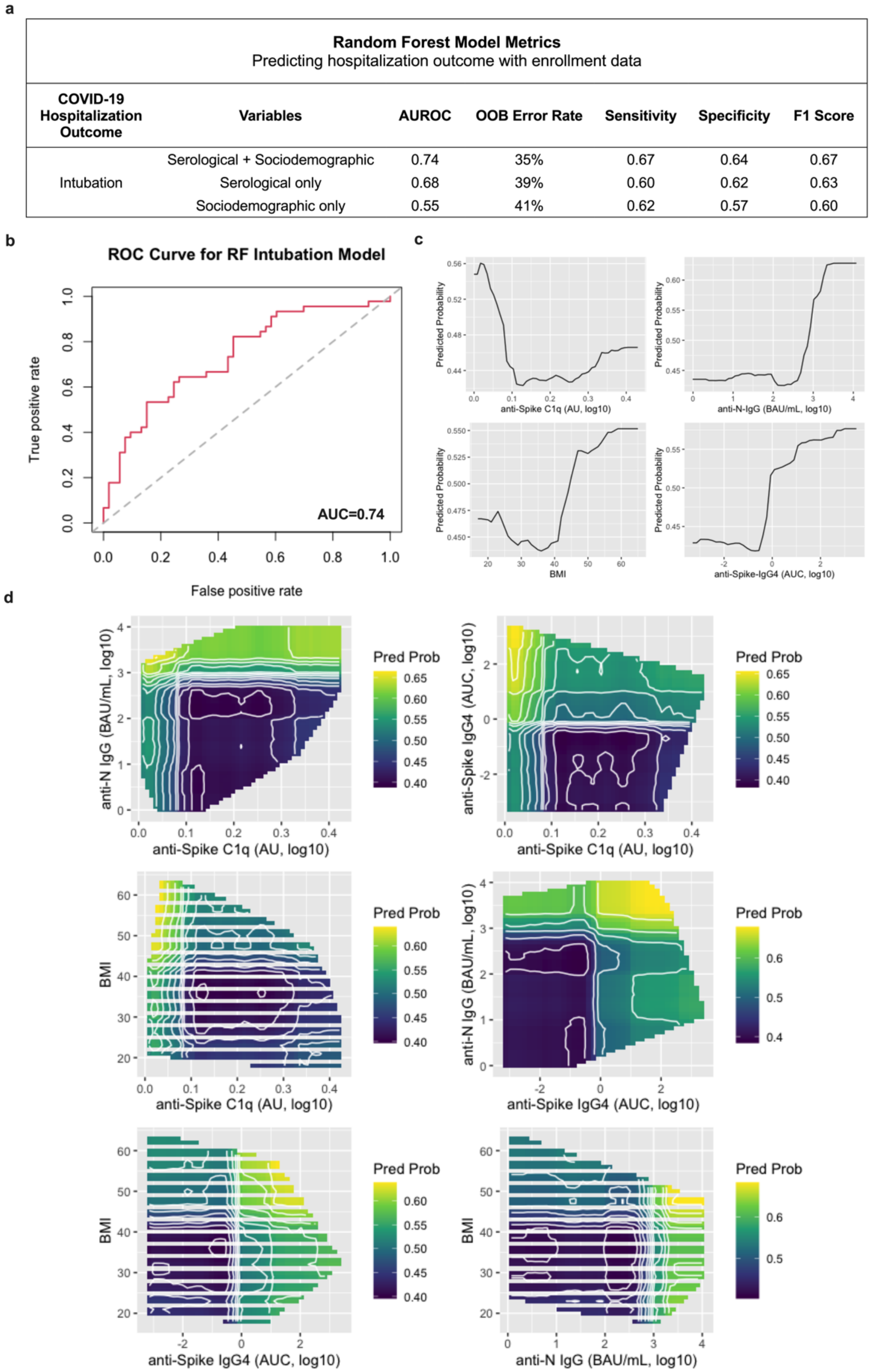

a

Random Forest Model Metrics
Predicting hospitalization outcome with enrollment data

COVID-19 Hospitalization Outcome	Variables	AUROC	OOB Error Rate	Sensitivity	Specificity	F1 Score
	Serological + Sociodemographic	0.74	35%	0.67	0.64	0.67
Intubation	Serological only	0.68	39%	0.60	0.62	0.63
	Sociodemographic only	0.55	41%	0.62	0.57	0.60

Supplementary Figure 10. Model performance metrics at a cutoff of 0.21 for the random forest death models at enrollment (n=21 subsequent deaths of 98 hospitalized with complete data). Receiver operating characteristic (ROC) curve for the random forest model with sociodemographic and serological measures with an area under the curve (AUC) value of 0.70. For the random forest model with sociodemographic and serological measures, partial dependence plots and bivariate dependence plots were used to assess the associations of the top four variables with the predicted probability of death among hospitalized COVID-19 patients, while controlling for all other variables.

a

Random Forest Model Metrics
Predicting hospitalization outcome with enrollment data

COVID-19 Hospitalization Outcome	Variables	AUROC	OOB Error Rate	Sensitivity	Specificity	F1 Score
	Serological + Sociodemographic	0.70	35%	0.67	0.65	0.75
Death	Serological only	0.68	35%	0.67	0.65	0.75
	Sociodemographic only	0.54	45%	0.38	0.60	0.68

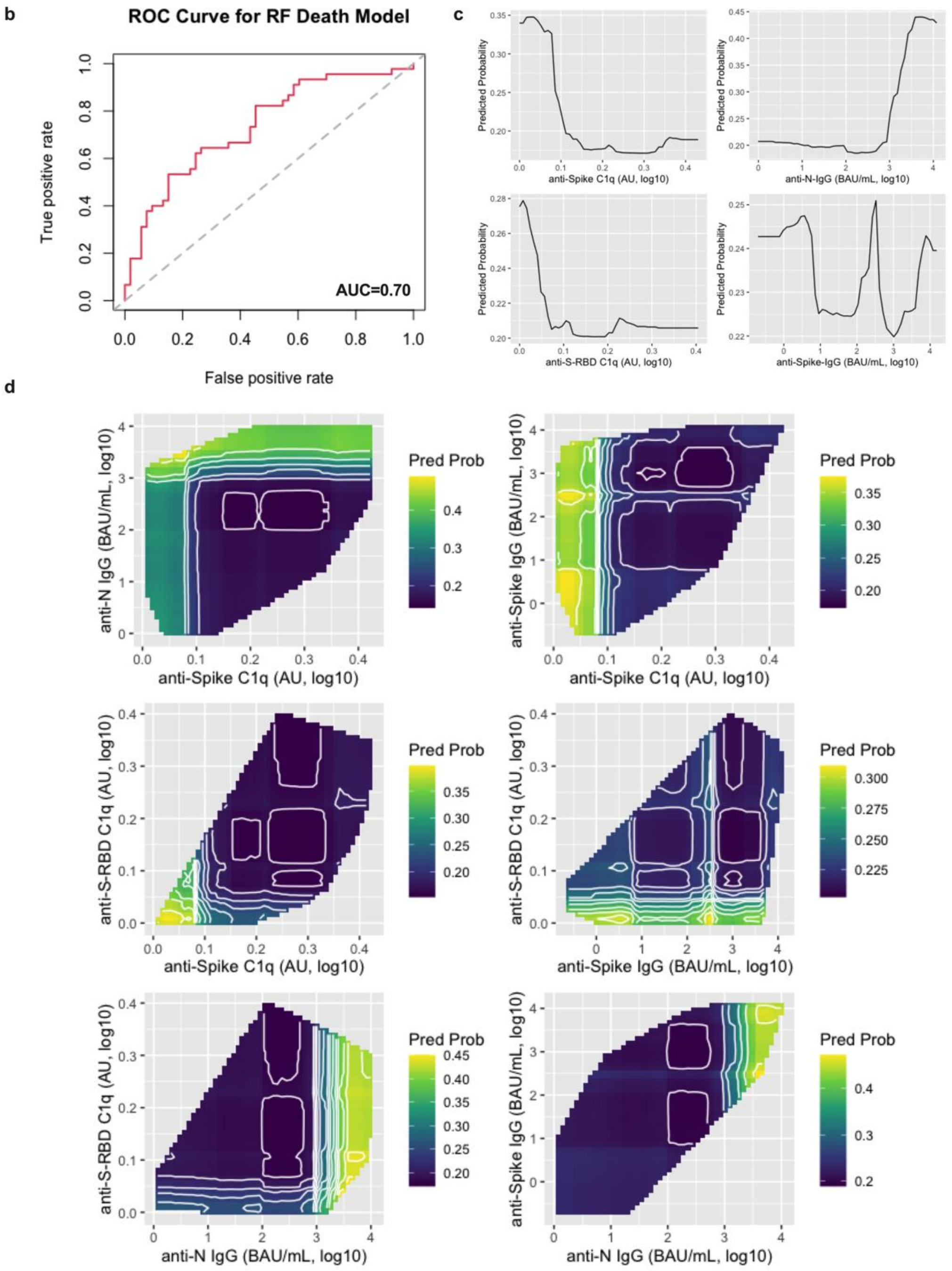

Supplementary Figure 11. Stratified 10-fold cross-validation random forest model performance metrics for classifying intubation (n=45 of 98 with complete data) and death (n=21 of 98 with complete data), including AUROC (area under the receiver operating characteristic), out-of-bag (OOB) error rates, specificity, and sensitivity. Variable importance plots for the models including both serological and sociodemographic data are shown. Complete datasets from enrollment for hospitalized patients were used for random forest modeling classification of intubation or death due to COVID-19.

a

Stratified 10-Fold Cross-Validation Random Forest Model Metrics
Predicting hospitalization outcome with enrollment data

COVID-19 Hospitalization Outcome	Variables	AUROC	OOB Error Rate	Sensitivity	Specificity
Intubation	Serological + Sociodemographic	0.78	30.6%	0.78	0.70
	Serological only	0.71	34.7%	0.67	0.60
	Sociodemographic only	0.62	44.9%	0.55	0.56
Death	Serological + Sociodemographic	0.75	18.4%	0.91	0.30
	Serological only	0.76	25.5%	0.86	0.35
	Sociodemographic only	0.61	22.5%	0.98	0.00

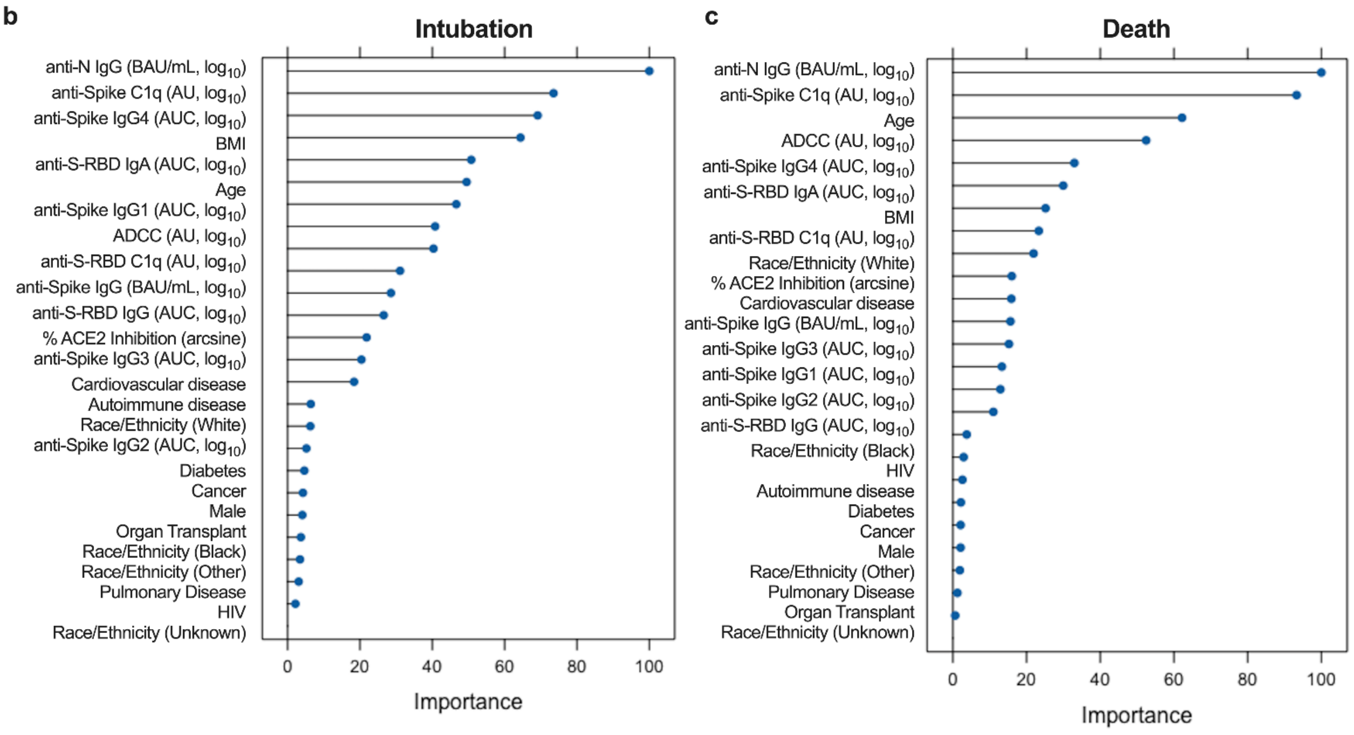

Supplement: Supplementary file 2 — Supplementary Tabels and Figures [file 43856_2024_658_MOESM2_ESM.pdf]
